# Hydrogels in the clinic: An update

**DOI:** 10.1002/btm2.10680

**Published:** 2024-05-16

**Authors:** John R. Clegg, Kolade Adebowale, Zongmin Zhao, Samir Mitragotri

**Affiliations:** ^1^ Stephenson School of Biomedical Engineering University of Oklahoma Norman Oklahoma USA; ^2^ Stephenson Cancer Center University of Oklahoma Health Sciences Center Oklahoma City Oklahoma USA; ^3^ Harold Hamm Diabetes Center University of Oklahoma Health Sciences Center Oklahoma City Oklahoma USA; ^4^ Institute for Biomedical Engineering, Science, and Technology University of Oklahoma Norman Oklahoma USA; ^5^ John A. Paulson School of Engineering and Applied Sciences Harvard University Allston Massachusetts USA; ^6^ Wyss Institute for Biologically Inspired Engineering at Harvard University Boston Massachusetts USA; ^7^ Department of Pharmaceutical Sciences, College of Pharmacy University of Illinois at Chicago Chicago Illinois USA; ^8^ University of Illinois Cancer Center Chicago Illinois USA

**Keywords:** clinic, clinical translation, clinical trials, crosslinking, drug delivery, hydrogel, injectable materials, tissue engineering, translational medicine

## Abstract

Hydrogels have been used in the clinic since the late 1980s with broad applications in drug delivery, cosmetics, tissue regeneration, among many other areas. The past three decades have witnessed rapid advances in the fields of polymer chemistry, crosslinking approaches, and hydrogel fabrication methods, which have collectively brought many new hydrogel products, either injectable or non‐injectable, to clinical studies. In an article published in 2020 entitled “Hydrogels in the clinic”, we reviewed the clinical landscape and translational challenges of injectable hydrogels. Here, we provide an update on the advances in the field and also extend the scope to include non‐injectable hydrogels. We highlight recently approved hydrogel products, provide an update on the clinical trials of injectable hydrogels, and discuss active clinical trials of topically applied and implantable hydrogels.

**Translational Impact Statement**

This review provide an update on the clinical landscape of injectable and non‐injectable hydrogels, highlighting medical products based on hydrogels that have been FDA/EMA approved or investigated in active clinical trials.

## 1 INTRODUCTION

Hydrogels remain one of the most widely studied technologies in the clinic with a wide spectrum of biomedical applications such as in drug delivery and tissue engineering. The clinical landscape of hydrogels continues to rapidly evolve, driven by the invention of new hydrogels, addition of new functionalities to existing hydrogels, approval of new hydrogel products, and the growing need of hydrogels in solving emerging clinical problems.

In a review article published in 2020,^1^ we reviewed the clinical landscape of injectable hydrogels, an important subset of hydrogels broadly used or investigated in the clinic. Our analysis revealed that the primary clinical applications of bulk hydrogel biomaterials were soft contact lenses (202 active trials in 2020), with topically applied hydrogel materials (e.g., wound dressings, 99 active trials in 2020), injectable hydrogels for tissue augmentation or regeneration (116 trials in 2020), and hydrogel‐based coils for endovascular embolization (8 trials in 2020) each being evaluated to a less frequent extent. Thirty‐six percent of clinically approved injectable hydrogels and 26% of clinical trials involving hydrogel products at that time incorporated a bioactive agent for drug delivery. Focusing our detailed analysis on a subset of hydrogel products—injectable hydrogels—we highlighted 28 injectable hydrogel‐based products approved by the US Food and Drug Administration (FDA) and/or European Medicines Agency (EMA). We detailed 31 active clinical trials investigating injectable hydrogels for regenerative medicine, cancer care, urinary incontinence, and ocular applications. We also summarized a set of biological, technological, and regulatory challenges that have limited the translation of injectable hydrogels.

As a 4‐year update to our last review, we extend our discussions to include both injectable and non‐injectable hydrogels, covering additional topics such as hydrogel‐based implants, topical treatments/wound dressings, and drug delivery devices. As a result of this expanded definition, we now highlight over 100 approved products and 210 active clinical trials of both injectable and non‐injectable hydrogels. This updated review highlights the widespread clinical studies of hydrogels in some important biomedical applications, in not only the prevailing applications of cosmetic surgery, musculoskeletal regenerative medicine, skin wound healing, optometry, and cancer treatment, but also the emerging areas of surgical adhesion prevention, implant‐associated infection treatment, transdermal drug delivery, female reproductive health, and male birth control. Collectively, this article provides an updated snapshot of the current clinical landscape of hydrogels in 2024.

## 2 CLARIFYING DEFINITIONS AND IMPORTANT CONSIDERATIONS FOR THERAPEUTIC HYDROGEL PRODUCTS

Here, we defined a “therapeutic hydrogel product” as a material comprised of crosslinked (physical, chemical, or otherwise) polymers, which is swollen in aqueous solution, and is designed to address a medical problem. Our working definition allowed us to properly refine our analysis, excluding viscous gel products, non‐aqueous gels, and products that are not indicated to treat diseases. Our analysis revealed that the backbone polymers for clinical hydrogels continue to be sourced from natural or synthetic sources, including a limited number of composites that combine natural and synthetic polymers. Only five polymers, hyaluronic acid (HA), silicone, poly(ethylene glycol) (PEG), collagen, and cellulose, account for more than half of the approved hydrogel products and more than half of the hydrogels in current clinical trials. The remaining products and trials use other biomaterials.

There are unique design considerations for hydrogel products, both approved and emerging.^2^, ^3^, ^4^ Hydrogel products are formed through crosslinking, covalent or physical, of hydrophilic polymers in aqueous solution. Hydrogels are suitable carriers for the encapsulation and delivery of diverse drugs or biologics, ranging from small molecule anesthetics, to hormones, whole proteins, and stem cells. Hydrogel products, distinctively from other classes of therapeutic biomaterials, are typically designed to integrate with nearby tissue, mimic tissue mechanical properties, support niches with biomimetic pH and ionic strength.^5^, ^6^ Therapeutic hydrogels participate in a dynamic interplay (i.e., infiltration, modification, and/or degradation) with the tissue surrounding their injection, implantation, or topical application.

The design considerations for hydrogel products pose a particular challenge for cumulative analyses on hydrogel products. Hydrogel device parameters, such as the polymer volume fraction of individual hydrogel backbone components, extent to which a backbone polymer is chemically modified, and the extent of crosslinking achieved are typically proprietary and rarely disclosed on approved product information sheets or clinical trial records. Other descriptive information on the hydrogels' individual polymeric components (e.g., polymer molecular weight and polydispersity), precursor rheology (e.g., viscosity), and crosslinked hydrogels (e.g., storage and loss modulus under shear, elastic modulus under compression, or tangent of the phase angle) are also not provided. The hydrogel dose or dose range for individual clinical trials is also often omitted, even when the precise dose of loaded drugs or therapeutics are provided. Limited availability of these important hydrogel design and delivery parameters limits otherwise fruitful opportunities for meta‐analysis, linking hydrogel design to clinical outcome. If these data were made available in the future, it could enable researchers to abstract new and important structure–function–outcome relationships for therapeutic hydrogels and human patients.

In our following summaries of approved and investigatory hydrogel products, we highlight key available aspects of each device (e.g., biomaterial, natural or synthetic material origin, route of administration, therapeutics delivered).

## 3 APPROVED PRODUCTS

Over 100 hydrogel products, derived from natural materials, synthetic materials, or their combination, have been approved by the FDA and/or EMA for tissue regeneration, tissue augmentation, facial correction, contact lens and other ocular applications, wound dressing, and as drug delivery devices. An updated list of approved hydrogels is shown in Table 1. Table 1 is a representative, but not exhaustive list, as there are many similar approved products in each category. We highlighted recently approved products.

**TABLE 1:** Approved hydrogel products grouped by broad indications and materials.

	Name (company)	Hydrogel material/payload	Administration method	Approved indication	Approval year
Injectable hydrogel products					
Facial correction: synthetic	Radiesse®(+) (Merz Pharmaceutical)^a^	Hydroxylapatite, carboxymethylcellulose with lidocaine	Dermis	Correction of wrinkles and folds, stimulation of natural collagen production	FDA (2015)
	Radiesse® (Bioform Medical, Inc.)^a^	Hydroxylapatite, carboxymethylcellulose	Dermis	Correction of facial folds and wrinkles, signs of facial fat loss and volume loss	EMA (2004), FDA (2006 for first indication)
	Artefill® (Suneva Medical, Inc.)^a^	Polymethylmethacrylate beads, collagen, and lidocaine	Dermis	Facial wrinkles and folds	FDA (2006)
	Bellafill® PMMA Collagen Dermal Filler (Suneva Medical, Inc.)	Polymethylmethacrylate beads, Bovine collagen, and lidocaine	Dermis	Wrinkles and acne scars	FDA (2006 for first indication)
	Ellansé (Sinclair)	Polycaprolactone (PCL) microspheres in carboxymethyl cellulose hydrogel	Dermis	Correction of wrinkles and folds, stimulation of natural collagen production	CE (2009)
Facial correction: natural	Belotero balance®(+) Lidocaine (Merz Pharmaceuticals)^a^	Hyaluronic acid with lidocaine	Dermis	Moderate to severe facial wrinkles and folds	FDA (2019)
	Revanesse® Versa, Revanesse® Ultra (Prollenium Medical Technologies, Inc.)^a^	Hyaluronic acid	Dermis	Moderate to severe facial wrinkles and folds	FDA (2017)
	Revanesse® Versa+ (Prollenium Medical Technologies, Inc.)^a^	Hyaluronic acid with lidocaine	Dermis	Moderate to severe facial wrinkles and folds	FDA (2018)
	Revanesse® Lips+ (Prollenium Medical Technologies, Inc.)	Hyaluronic acid	Lips	Restore lost volume and create a fuller‐looking lip	FDA (2020)
	Teosyal® RHA (Teoxane SA)^a^	Hyaluronic acid	Dermis	Facial wrinkles and folds	EMA (2015), FDA (2017)
	Restylane® Lyft, Restylane® Refyne, Restylane® Defyne (Galderma Laboratories, L.P.)^a^ Restylane® Silk (Valeant Pharmaceuticals North AmericaLLC/Medicis)^a^ Restylane® Injectable Gel (Medicis Aesthetics Holdings, Inc.)^a^ Restylane® Kysse, Restylane® Eyelight (Galderma Laboratories, L.P.)	Hyaluronic acid with lidocaine	Subcutaneous, dermis, lips	For correction of volume deficit, facial folds and wrinkles, midface contour deficiencies, perioral rhytids, and infraorbital hollowing	EMA (2010), FDA (2012 for first indication, 2023 for last indication)
	Belotero balance® (Merz Pharmaceuticals)^a^	Hyaluronic acid	Dermis	Moderate to severe facial wrinkles and folds	EMA (2004), FDA (2011 for first indication)
	Juvéderm® XC (Allergan, Inc.)^a^	Hyaluronic acid with lidocaine	Facial tissue	Correction of facial wrinkles and folds	FDA (2010)
	Evolence® Collagen Filler (Colbar Lifescience)^a^	Collagen	Dermis	Moderate to deep facial wrinkles and folds	EMA (2004), FDA (2008)
	Elevess® (Anika Therapeutics)^a^	Hyaluronic acid with lidocaine	Dermis	Moderate to severe facial wrinkles and folds	FDA (2006), EMA (2007)
	Juvéderm® Voluma XC/Ultra XC/Volbella XC/Vollure XC/VOLBELLA XC/VOLUX XC (Allergan, Inc.)^a^	Hyaluronic acid	Facial tissue, cheek, lips	For correction of facial wrinkles and folds, volume loss, lip augmentation, undereye hollows, and jawline definition	EMA (2000), FDA (2006 for first indication, 2022 for last indication)
	Hylaform® (Hylan B gel), Captique Injectable Gel, Prevelle Silk (Genzyme Biosurgery)^a^	Modified hyaluronic acid derived from a bird (avian) source	Dermis	Correction of moderate to severe facial wrinkles and folds	EMA (1995), FDA (2004)
	Collagen Implant, CosmoDerm® 1 human‐based collagen, CosmoDerm® 2 human‐based collagen CosmoPlast® human‐based collagen (Inamed Corporation/Allergan, Inc.)^a^	Human collagen	Superficial papillary dermis	For correction of soft tissue contour deficiencies, such as wrinkles and acne scars	FDA and EMA (2003)
	Fibrel® (Serono Laboratories)^a^	Collagen	Dermis	For correction of depressed cutaneous scars	FDA (1988)
	Zyplast(R)® and Zyderm(R)® (Inamed Corporation/Allergan, Inc.)^a^	Bovine collagen	Dermis	For correction of contour deficiencies	FDA and EMA (1981)
	RHA® 2, 3, 4, Redensity (Teoxane S.A.)	Hyaluronic acid, 1,4‐butanediol diglycidyl ether with lidocaine	Dermis	Correction of moderate to severe dynamic perioral rhytids	FDA (2017 for first indication, 2021 for last indication)
	SKINVIVE by JUVÉDERM (Allergan)	Hyaluronic acid, 1,4‐butanediol diglycidyl ether with lidocaine	Intradermal	Improve the smoothness of the cheeks	FDA (2023)
Tissue fusion, repair and regeneration: synthetic	Emdogain (Straumann)	Porcine enamel matrix derivative in propylene glycol alginate gel	Flap incision or flapless injection	Regenerates periodontal tissue (cementum, periodontal ligament, bone)	FDA (1996)
	PerioGlas (NovaBone)	Calcium phosphosilicate particles, a PEG and glycerine gel‐like binder	Injection	Dental bone regeneration	FDA (2005)
	Actifuse (Baxter)	Phase‐pure silicon‐substituted calcium phosphate in poloxamer 407	Injection	Bone void filler in spinal and orthopedic application	FDA (2018)
	Dynagraft II (IsoTis Orthobiologics)	Demineralized bone matrix in poloxamer	Injection	Bone void filler	FDA (2005)
	AlloFuse Plus Paste, AlloFuse Plus Putty (AlloSource)	Allographic demineralized bone matrix in polyethylene oxide polypropylene oxide block copolymer	Spinal fusion or injection to trauma area	Void filler, graft extender	FDA (2011)
	Optium DBM Gel (LifeNet Health)	Allographic demineralized bone matrix in glycerol	Spinal fusion or injection to trauma area	Bone graft extender and void filler	FDA (2005)
	Grafton DBM gel (Medtronic)	Allographic demineralized bone matrix in glycerol	Spinal fusion or injection to trauma area	Bone graft extender and void filler	FDA (2005)
	Arthrosamid (Contura International)	Polyacrylamide	Intraarticular	Treatment of pain in osteoarthritis (OA)	CE(2007), FDA (2014)
	GelrinC® (Regentis Biomat.)	PEG diacrylate with denatured fibrinogen	Intraarticular injection after microfracture	Treatment of focal cartilage lesions	CE (2017)
Tissue fusion, repair and regeneration: natural	INFUSE® bone graft (Medtronic Sofamor Danek USA, Inc.)^a^	Collagen and recombinant human bone morphogenetic protein‐2	Spinal injection	Spinal fusion, and spine, oral‐maxillofacial and orthopedic trauma surgeries	FDA (2002 for first indication)
	Osteogenic protein 1 (OP‐1®) implant, OP‐1® Putty (Stryker Biotech)^a^	Collagen, carboxymethylcellulose, and recombinant OP‐1	Spinal injection	Posterolateral lumbar spinal fusion	FDA (2001)
	Algisyl‐LVR® Hydrogel Implant (LoneStar Heart, Inc.)^a^	Alginate	Percutaneous	Advanced heart failure	EMA (2014)
	Tactoset (Anika Therapeutics)	Hyaluronic acid with calcium phosphate	Injection	Bone void filler for orthopedic application, augmentation of hardware and support of bone fragments during surgery	FDA (2019 for first indication)
	Kinex Bioactive Gel (Globus Medical)	Bioglass, collagen and hyaluronic acid	Injection	Bone void filler	FDA (2013)
	EUFLEXXA® (Ferring Pharmaceuticals, Inc.)^a^	Hyaluronic acid	Intra‐articular	Treatment of pain in osteoarthritis (OA)	FDA (2004), EMA (2005)
	Gel‐One (Zimmer Biomet)	Cinnamic acid functionalized hyaluronic acid	Intraarticular injection	Treatment of pain in osteoarthritis (OA)	FDA (2011)
	Monovisc (Anika Therapeutics)	High MW hyaluronic acid lightly crosslinked with biscarbodiimide	Intraarticular injection	Treatment of pain in osteoarthritis (OA)	CE (2007), FDA (2014)
	Cingal (Anika Therapeutics)	High MW hyaluronic acid lightly crosslinked with biscarbodiimide and triamcinolone hexacetonide	Intraarticular injection	Treatment of pain in osteoarthritis (OA)	CE (2016)
	Hymovis (Fidia Farmaceutici)	Hyaluronic acid 500–730 kDa, functionalized with 2%–3% hexadecylamine	Intraarticular injection	Treatment of pain in osteoarthritis (OA)	FDA (2015)
	TRIVISC (Orthogenrx, Inc.)	Hyaluronic acid	Intraarticular injection	Treatment of pain in osteoarthritis (OA)	FDA (2017)
	SINOVIAL (IBSA INSTITUT BIOCHIMIQUE SA)	Hyaluronic acid	Intraarticular injection	Treatment of pain in osteoarthritis (OA)	FDA (2014)
	SYNOJOYNT (Arthrex, Inc.)	Hyaluronic acid	Intraarticular injection	Treatment of pain in osteoarthritis (OA)	FDA (2018)
	Orthovisc (Anika Therapeutics)	Hyaluronic acid	Intraarticular	Treatment of pain in osteoarthritis (OA)	FDA (2004)
	BST‐CarGel® (Smith & Nephew)	Chitosan	Mini‐arthrotomy or arthroscopy	Cartilage repair	CE (2012)
Drug delivery device: synthetic	Jelmyto (UroGen Pharma)	Pluronic F‐127, PEG‐400, HPMC/mitomycin	Catheter instillation	Low‐grade upper tract urothelial cancer	FDA (2020)
	Vantas® (Endo Pharmaceuticals)^a^	Histrelin acetate, poly(2‐hydroxyethyl methacrylate), poly(2‐hydroxypropylmethacrylate) and gonadotropin releasing hormone	Subcutaneous	Palliative treatment of prostate cancer	FDA (2004), EMA (2005)
Others	TraceIT® Hydrogel Tissue Marker (Augmenix, Inc.)^a^	Polyethylene glycol	Percutaneous	Improved soft tissue alignment for image‐guided therapy	FDA (2013)
	Supprelin LA® (Indevus Pharmaceuticals, Inc.)^a^	Histrelin acetate, poly(2‐hydroxyethyl methacrylate)	Subcutaneous	Central precocious puberty	EMA (2005), FDA (2007)
	Bulkamid® hydrogel (Searchlight Pharma)^a^	Polyacrylamide	Transurethral	Female stress urinary incontinence	EMA (2003), FDA (2006)
	Coaptite® (BioForm Medical, Inc.)^a^	Calcium hydroxylapatite, sodium carboxymethylcellulose, glycerin	Submucosal	Female stress urinary incontinence	EMA (2001), FDA (2005)
	SpaceOAR® Hydrogel (Augmenix, Inc.)^a^	Polyethylene glycol	Percutaneous	For protecting vulnerable tissues during prostate cancer radiotherapy	EMA (2010), FDA (2015)
	Solesta® (Oceana Therapeutics, Inc.)	Hyaluronic Acid (NASHA) and dextranomer (Dx)	Injection	Fecal incontinence	FDA (2011)
Non‐injectable hydrogel products					
Drug delivery device: synthetic	Cervidil (Ferring Laboratories)	PEG 8000, dicyclohexyl methane‐4, 4′‐diisocyanate and 1,2,6‐hexanetriol/dinoprostone	Vaginal insert	Initiation and/or continuation of cervical ripening in pregnant women at or near term	FDA (1993)
	Zuplenz TM (Galena Bipharma)	PEG 1000, polyvinyl alcohol and rice starch/Ondansetron	Oral gel	Chemotherapy, radiation, and postoperative‐induced nausea and vomiting	FDA (1991)
Tissue repair: natural	Apligraf (Organogenesis)	Collagen with fibroblast and keratinocytes	Petroleum jelly–impregnated gauze	Diabetic foot ulcer and venous leg ulcers	FDA (2000)
	GRAFTJACKET Now (Wright Medical Group)	Collagen with different cells	Surgical implant	Tendon and ligamentous tissue repair	FDA (2010)
	OrthADAPT (Synovis Orthopedic and Woundcare)	Collagen with different cells	Implant	Attaching tissue to bone, tendon repair	FDA (2005)
	Permaco (Covidien)	Collagen	Implant	Tendon and ligamentous repair, surgical implant for ventral hernia repair and abdominal wall reconstruction	FDA (2005)
	TissueMend (Stryker)	Collagen	Implant	Tendon and ligamentous repair	FDA (2006)
	Zimmer Collagen Repair Patch (Tissue Science Laboratories)	Collagen	Implant	Rotator cuff and tendon repair	FDA (2006)
	OrthADAPT® Bioimplant (Pegasus Biologics)	Collagen	Surgical mesh	Attachment of tissue to bone, tendon repair	FDA (2012)
	TissueMend® (TEI Biosciences)	Collagen	Implant	Tendon and ligament repair	FDA (2002)
	Zimmer® Collagen Repair Patch (Zimmer Biomet)	Collagen	Implant	Rotator cuff and tendon repair	FDA (2002)
	Permacol® (Medtronic)	Porcine decellularized matrix	Implant	Tendon and ligament repair, surgical implant for ventral hernia repair and abdominal wall reconstruction	FDA (1999)
Tissue repair: synthetic	DuraSeal (Duraseal)	PEG ester, trilysine amine, decahydrated sodium borate, and others	Surgical implant	Prevention of cerebrospinal fluid leakage after cranial and spinal surgery	FDA (2005)
Contact lenses and other ocular applications: synthetic	Airsoft™ (Maxvue Vision)	Silicone	Contact lens	Astigmatism	CE (2009)
	Abiliti Overnigh Therapeutic Lenses, ACUVUE OASYS (Johnson & Johnson)	Silicone	Contact lens	Myopia and/or hyperopia	FDA (2021); FDA (2018)
	Clariti 1 day (Cooper Vision)	Silicone	Contact lens	Short sight and long sight	FDA (2018)
	MiSight 1 Day (BenQ)	Omafilcon A	Contact lens	Myopia	FDA (2019)
	Dailies AquaComfort (Ciba Vision)	Nelfilcon A	Contact lens	Astigmatism; optical correction of refractive ametropia	FDA (2018)
	Proclear (Omafilcon B) (Cooper Vision)	2‐Hydroxyethylmethacrylate and 2‐methacryloxyethyl phosphorylcholine, ethylene glycol dimethacrylate	Contact lens	Daily wear for correction of visual acuity	FDA (2013)
	Resure Sealant (Ocular Therapeutix)	PEG	Topical	Intraoperative management of clear corneal incisions following cataract surgery	FDA (2014)
	Restasis (Allergan)	Carbomer copolymer type A with cyclosporine	Topical/eye drop	Increase tear production	FDA (2002)
	Yutiq (EyePoint Pharmaceuticals)	Polyvinyl alcohol with fluocinolone acetonide	Intravitreal implant	Chronic non‐infectious uveitis affecting the posterior segment of the eye	FDA (2018)
	Ozurdex (Allergen)	PLGA with dexamethasone	Intravitreal implant	Macular edema, non‐infectious uveitis	FDA (2009)
	Iluvien (Alimera Sciences)	Polyvinyl alcohol, silicone adhesives with fluocinolone acetonide	Intravitreal implant	Diabetic macular edema	FDA (2014)
	Azasite (Merck)	Poloxamer407, polycarbophil with azithromycin	Topical/eyedrop	Bacterial conjunctivitis	FDA (2007)
	BromSite (Sun Pharmaceutical Industries, Inc.)	Poloxamer407, polycarbophil with bromfenac sodium sesquihydrate	Topical/Ocular instillation	Postoperative inflammation and prevention of ocular pain in patients undergoing cataract surgery	FDA (2016)
	Besivance (Bausch + Lomb)	Poloxamer407, polycarbophil with besifloxacin	Topical/Ocular instillation	Bacterial conjunctivitis	FDA (2009)
	Bausch + Lomb ULTRA (Bausch + Lomb)	Samfilcon A	Contact lens	Near‐sightedness, far‐sightedness, and astigmatism	FDA (2018)
	SBL‐3 Multifocal Intraocular Lens (Lenstec, Inc.)	Hydrophilic acrylic	Intraocular	Vision correction after the eye's natural lens is removed because of cataract	FDA (2022)
	Precision7; Precision7 for Astigmatism; Precision7 Multifocal; Precision7 Multifocal Toric (Alcon)	Senofilcon A	Contact lens	Myopia, hyperopia, astigmatism	FDA (2023)
	Timoptic XE (Bausch + Lomb)	Anionic heteropolysaccharide with timolol maleate	Topical/eyedrop	Elevated intraocular pressure in patients with ocular hypertension or open‐angle glaucoma	FDA (1993)
	EVO ICL and EVO TICL (STAAR Surgical Company)	Collamer	Intraocular	Astigmatism and nearsighted	FDA (2022)
	Visian® Toric ICL (STAAR Surgical Company)	Collamer	Intraocular	Myopia and astigmatism	FDA (2018)
Wound dressing: synthetic	3 M™ Tegaderm™ hydrogel wound filler (3 M)	Propylene glycol	Dermal wound filler	Low to moderate draining wounds, partial and full‐thickness dermal ulcers	FDA (2018)
	Cutimed gel (BSN Medical)	Carbomer 940	Wound dressing	Management of dry to low exuding wounds	FDA (2014)
	Woun'Dres (Coloplast)	Carbomer, collagen	Wound dressing	Dry wounds	FDA (1999)
	DermaSyn (DermaRite Industries)	Carbomer940	Wound dressing	Management of acute or chronic partial and full thickness wounds/ulcers that are dry or have minimal exudate	FDA (2006)
	Suprasorb G (Lohmann & Rauscher Global)	CMC polymer, propylene glycol	Bandage	Dry wounds, lower leg ulcer, pressure ulcer, first and second‐degree burns, scalds	FDA (2016)
	DermaGauze™ (DermaRite industries)	Acrylate polymer	Hydrogel impregnated gauze	Acute or chronic partial and full thickness wounds	FDA (2014)
	Simpurity™ Hydrogel (Safe n'Simple)	Polyethylene oxide, polyvinyl alcohol, acrylate, polyurethane	Absorben sheet	Dry wounds, skin burns and dry scabs	FDA (2011)
	AquaDerm™ (DermaRite industries)	2‐Acrylamido‐2 methyl‐1‐propanesulfonic acid sodium, propylene glycol, poly (ethylene glycol) dimethacrylate, 2‐hydroxy‐2‐methylpropiophenone	Hydrogel sheet	Pressure ulcers, minor burns and radiation tissue damage	FDA (2013)
Wound dressing: natural	Hyalofill (Anika)	Hyaluronic acid	Wound care and treatment	Absorb wound exudate, promote granulation tissue formulation, supports healing process	FDA (1999)
	HemCon bandage (HemCon Medical Technologies)	Chitosan	Bandage	Provide hemostatsis and antibacterial barrier	FDA (2003)
	Regenecare wound gel (MPM Medical)	Collagen, aloe and sodium alginate with lidocaine	Wound filling	Management of skin wounds	FDA (2002)
	Purilon® Gel (Coloplast)	Sodium carboxymethylcellulose, calcium alginate	Wound filling	Dry and sloughy necrotic wounds as well as wounds with a mix of necrotic and granulated tissue	FDA (1997)
	Prontosan wound gel (B. Braun Medical)	Glycerol, hydroxyethyl cellulose with polyhexamethylene biguanide and undecylenamidopropyl betaine	Would gel	Management of ulcers, burns, partial and full thickness wounds, large surface area wounds and surgical incisions	FDA (2013)
	Algicell Ag calcium alginate dressing with antimicrobial silver (Integra Life Science)	Calcium alginate with silver	Wound dressing	Abrasions and minor lacerations, cuts, scalds and burns	FDA (2008)
	INTRASITE gel hydrogel wound dressing (Smith & Nephew Healthcare)	Modified carboxymethyl cellulose, propylene glycol	Wound dressing	Re‐hydrates necrotic tissue, facilitating autolytic debridement, loosen and absorb slough and exudate	FDA (1990)
	SoloSite Gel (Smith & Nephew Healthcare)	Sodium carboxymethylcellulose	Wound dressing	Minor burns, superficial lacerations, cuts and abrasions (partial thickness wounds), skin tears, venous ulcers (leg ulcers), surgical incisions, diabetic foot ulcers	FDA (1998)
	NU‐GEL™ (Systagenix)	Sodium alginate	Wound dressing	Chronic wounds	FDA (1998)
	ActivHeal Hydrogel (Advanced Medical Solutions)	Alginate	Wound dressing	Rehydrates dry necrotic wounds	FDA (2001)
	Surgical Silver Post Operative Dressing (Advanced Medical Solutions)	Alginate with silver	Wound dressing	Post‐operative surgical wounds	FDA (2018)
Tissue augmentation: synthetic	IDEAL IMPLANT Saline‐filled Breast Implant (Ideal Implant)	Silicone	Implant	Breast implant	FDA (2014)
	MENTOR® MemoryShape® Breast Implants (J&J MedTech)	Silicone	Implant	Breast implant	FDA (2013)
	Natrelle 410 Highly Cohesive Anatomically Shaped Silicone‐Filled Breast Implant (Allergan)	Silicone	Implant	Breast implant	FDA (2013)
	Sientra Silicone Gel Breast Implants (Sientra, Inc.)	Silicone	Implant	Breast implant	FDA (2013)
Others	ReSure sealent (Ocular Therapeutix)	PEG	Topical	Intraoperative management of clear corneal incisions	FDA (2014)
	Progel™ Pleural Air Leak Sealant (NEOMEND, Inc.)	Human serum albumin (HSA) and polyethylene glycol (PEG)	Surgical implant	Seal air leaks in both open and minimally invasive thoracic surgery	FDA (2010)
	Plenity® (Gelesis, Inc.)	Cellulose and citric acid	Oral	Overweight, obesity	FDA (2019)

^a^ Products that were included in the 2020 “Hydrogels in the clinic” review.

Abbreviations: CE, Conformité Européenne mark in the European Union; EMA, European Medicines Agency; FDA, Food and Drug Administration; HPMC, hydroxypropyl methyl cellulose; PEG, poly(ethylene glycol); PLGA, poly(lactic‐co‐glycolic acid).

### 3.1 New injectable hydrogel products

Approved injectable hydrogels cover multiple applications including facial correction, tissue repair and regeneration, drug delivery formulations/devices, among others. Consistent with our 2020 data,^1^ injectable hydrogel‐based dermal fillers for facial correction remain a major area for approved products. These products are mainly formulated from natural polymers (e.g., HA and collagen) although synthetic materials (e.g., poly(lactic‐co‐glycolic acid) (PLGA), polymethylmethacrylate, carboxymethylcellulose) are also used in a few approved products. These products are typically injected to the dermis or subcutaneous space to correct winkles, folds, scars, or defective facial tissues. Since 2020, 6 new approvals have extended indications for an already‐approved product (RHA® Redensity, Restylane® Kysse, Restylane® Eyelight, Juvéderm® VOLBELLA XC, Juvéderm® VOLUX XC, SKINVIVE by Juvéderm®). These new approvals each indicate a previously approved hydrogel product for injection into a different facial site. For example, the approval of Restylane® Eyelight by the FDA in 2023 expanded the use of the Restylane® to include correction of infraorbital hollowing. Restylane® Eyelight was previously approved for correction of volume deficit, facial wrinkles and folds, and midface contour deficiency.^1^

#### 3.1.1 Tissue fusion, tissue repair, and tissue regeneration

Regenerative hydrogel products use injectable hydrogels to replace, repair or regenerate damaged, defective, or degenerated tissues. Approved hydrogel products or devices function as void fillers (e.g., Tactoset®, Grafton™ DBM), tissue fusion materials (e.g., INFUSE® bone graft, OP‐1® Putty), and visco‐supplements (SynoJoynt®, Arthrosamid®). Both synthetic (e.g., poloxamer, polyacrylamide, poly(ethylene glycol diacrylate) (PEGDA)), and natural polymers (e.g., HA, collagen, and chitosan) are extensively used in these productions with HA being the most common. While natural polymer‐based products afford advantages such as validated safety profiles and low immunogenicity, synthetic polymer‐based products may achieve better tunability by rational polymer design for desired hydrogel properties. Among them, one product that received FDA approval recently is Actifuse developed by Baxter. Actifuse is made from phase‐pure silicon‐substituted calcium phosphate in poloxamer 407 and was approved as bone void filler in spinal and orthopedic applications.^7^

#### 3.1.2 Drug delivery devices

Drug eluting hydrogel products use synthetic polymers. Two recently approved drug delivery formulations based on injectable synthetic hydrogels are Vantas and Jelmyto. Jelmyto, a hydrogel made from Pluronic F‐127, PEG‐400, and hydroxypropyl methyl cellulose (HPMC) for the delivery of mitomycin, received FDA approval in 2020 for the treatment of low‐grade upper tract urothelial cancer.^8^ Vantas is a hydrogel depot comprised of hydroxyethyl methacrylate and hydroxypropyl methacrylate, crosslinked with trimethylolpropane trimethacrylate, which releases histrelin acetate for 12 months into the upper arm (following subcutaneous injection) for palliative treatment of advanced prostate cancer. Injectable hydrogels have also been approved for other applications and some of them include urinary incontinence (e.g., Bulkamid®), fecal incontinence (Solesta®), central precocious puberty (Supprelin LA®), protecting vulnerable tissues during radiotherapy (SpaceOAR®), among others.

### 3.2 Non‐injectable hydrogels

Unlike injectable hydrogels, non‐injectable hydrogels must be administered as a surgical implant, applied topically, or inserted non‐invasively with medical devices. Hydrogel dosage forms or formulations that are used for wound dressings, medical implants, and contact lens often use synthetic, non‐biodegradable, and/or mechanically robust materials (e.g., silicone, poloxamers, or carbomers) that are used less frequently for hydrogel injections. However, the approved non‐injectable hydrogels share some common applications with approved injectable hydrogels such as drug delivery, tissue repair, and tissue regeneration.

#### 3.2.1 Bulk hydrogels as regenerative implants, tissue sealants, and delivery systems for pain management

Most bulk hydrogel products for implantation and drug delivery are made from synthetic polymers such as PEG and poly(vinyl alcohol) (PVA). Most approved non‐injectable hydrogels for tissue repair use natural polymers (collagen in particular) as the backbone material. These hydrogels are typically implanted or inserted, rather than injected, for repair of diabetic foot ulcer (e.g., Apligraf®), tendon and ligamentous tissue (e.g., GRAFTJACKET Now, Permaco, TissueMend®, Permacol®), rotator cuff repair (Zimmer® Collagen Repair Patch), and other applications. DuraSeal is an approved non‐injectable hydrogel derived from a synthetic polymer for tissue repair. DuraSeal is comprised of PEG ester, trilysine amine, and decahydrated sodium borate. DuraSeal is indicated for the prevention of cerebrospinal fluid leakage after cranial and spinal surgery.^9^ Hydrogels have further been used to promote regeneration of diverse tissues such as bone, tendon, ligament, and for the treatment of osteoarthritis. For example, a recent study showed that intradiscal injection of ReGelTec's Hydrafil hydrogel relieved chronic lower back pain caused by degenerative disc disease. Patients' pain level decreased from 7.1 to 2.0 (on a scale of 10) and they also experienced greatly improved physical function.^10^ These findings led to designation as an FDA Breakthrough Device.

#### 3.2.2 Bulk hydrogel contact lenses

Most of the approved hydrogel‐based contact lenses or ocular formulations use synthetic polymer‐based hydrogel such as silicone, nelfilcon A, carbomer, PEG, PVA, or poloxamer, although natural polymers such as HA and collamer are also used in some products. These products cover multiple ocular indications. Noted examples include astigmatism, myopia, hyperopia, drug eye, uveitis, macular edema, and glaucoma. Abiliti Overnight Therapeutic Lenses, Precision 7 family, and EVO TICL are three examples of recently approved hydrogel‐based contact lenses. For instance, the Precision 7 family including Precision 7, Precision 7 for Astigmatism, Precision 7 Multifocal, and Precision7 Multifocal Toric are Senofilcon A based soft contact lenses that are indicated for ocular indications including myopia, hyperopia, and astigmatism.

#### 3.2.3 Hydrogel wound dressings

Hydrogel wound dressings are another important class of approved non‐injectable hydrogel products, among which both synthetic and natural hydrogels are extensively used. Skin is the largest organ, provides an important barrier, and serves in immune functions.^11^ Compared to other organs, skin is relatively accessible and can typically be accessed in a minimally invasive manner. While we already described hydrogels for aesthetic dermal applications, other hydrogel products have been formulated for topical application and promotion of wound healing. For example, Apligraf® is an FDA‐approved product to treat chronic wounds such as diabetic foot ulcers and venous leg ulcers.^12^ This product can also be readily reapplied as needed. These approved wound dressing products are applied to the wound through diverse formats such as bandage, hydrogel sheet, gauze, and would‐filling gel to promote wound healing.

#### 3.2.4 Bulk hydrogel‐based surgical products

Additional surgical applications utilize approved hydrogel products. Silicone‐based hydrogels are used in plastic surgery, surgical reconstruction, and as tissue sealants.

## 4 CLINICAL TRIALS

**FIGURE 1:**
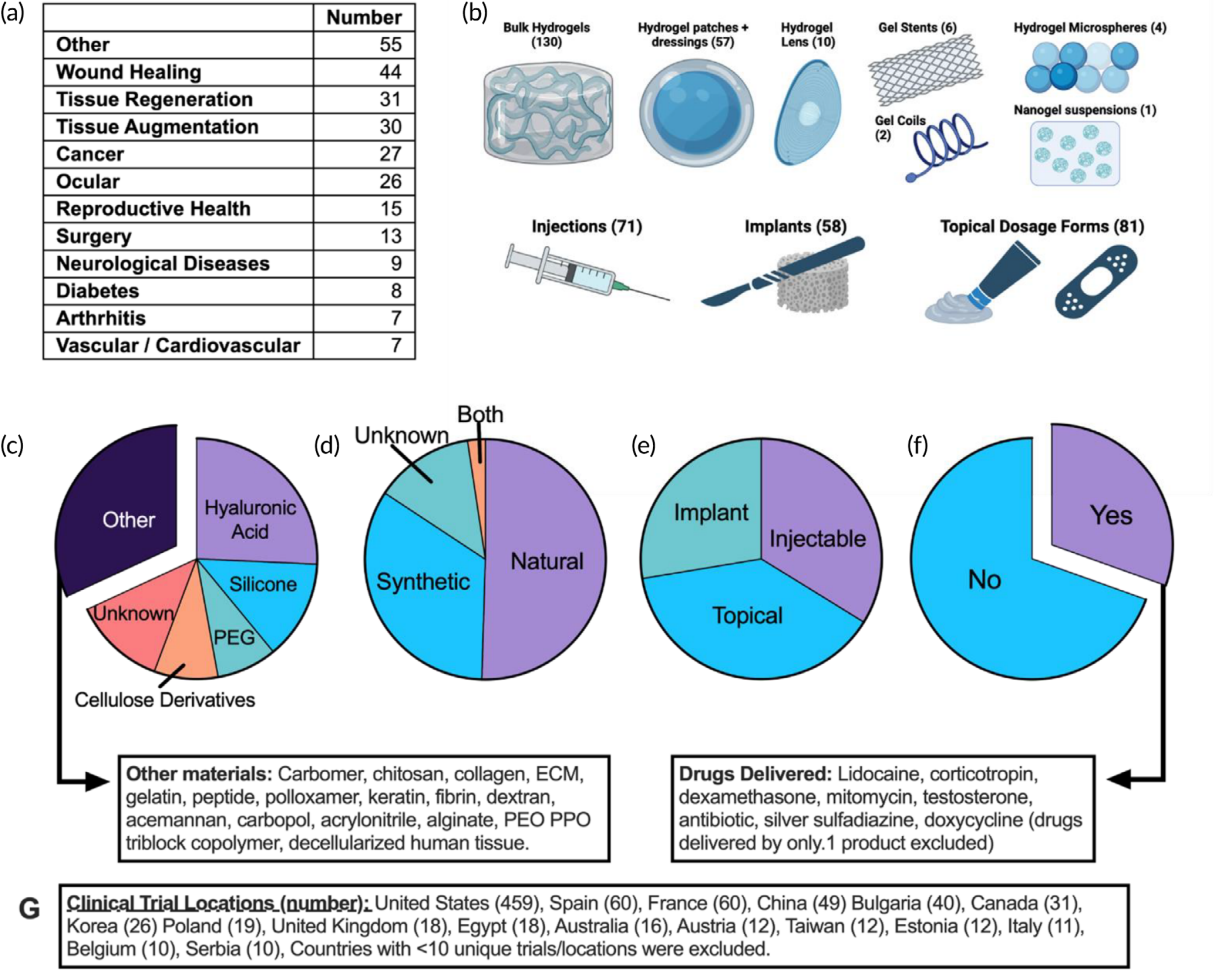
Overview of current hydrogel clinical trials. (a) Table summary of the cumulative number of clinical trials per indication. (b) Summary of hydrogel dosage forms, with the cumulative number of trials and a representative depiction. Graphic generated using BioRender. (c–f) Part‐of‐whole analysis summarizing the biomaterials, material origin, dosing method, and drug delivery of hydrogel clinical trials. (g) Summary of global clinical trial sites. The total number of sites is much more than 210, as single clinical trials often have many trial sites/locations. Data collected from ClinicalTrials.gov and are current as of June 20, 2023.

A comprehensive analysis of current hydrogel clinical trials was performed in the ClinicalTrials.gov database. Active, recruiting, and enrolling trials were included, with completed, terminated, suspended, or withdrawn trials excluded. An initial collection of potentially admissible clinical trials was collected by searching for “hydrogel” in trial intervention (80 trials), “hydrogel” as a search term (89 trials), “gel” as an intervention (518 trials) and “extracellular matrix” as an intervention (24 trials). Once duplicate returns were removed, our dataset contained 632 active “gel”, “hydrogel”, or “ECM” trials. The dataset was refined further by excluding observational or non‐interventional clinical trials, excluding trials where the hydrogel was not an active component of the intervention (e.g., where a gel pad was used as a dressing in a medical device trial), and excluding any trial where the intervention did not meet the working definition of a hydrogel (i.e., crosslinked polymers swollen in an aqueous solution). The resulting dataset contained 210 unique hydrogel clinical trials. A content analysis was performed, where each trial was categorized by the hydrogel form (i.e., bulk material, patch or dressing, particulate suspension, lens, or coil), disease indicated, material origin (i.e., natural, synthetic, both, or unknown), and whether an active agent was delivered. Product names, biomaterial compositions, and specific drugs delivered were also noted. A summary of these data is presented in Figure 1.

### 4.1 Clinical trial overview and update

Tissue regeneration, soft tissue augmentation, cancer, urinary incontinence, and related indications have persisted as leading applications for hydrogel products, with approximately 100 unique current clinical trials and a similar number of unique products. Our expanded scope for data collection led to identification of wound healing products as the most common application for hydrogel products in current clinical trial. Our analysis also revealed new biomaterials (i.e., no active trials with the material composition in 2020) and new applications (i.e., new clinical trial indications for hydrogel therapeutics that have no associated approved products and were not captured in our 2020 analysis).

### 4.2 An update on injectable hydrogel clinical trials

Four injectable hydrogel products that were captured in our 2020 analysis have ongoing clinical trials in 2024. (Algisyl‐LVR for intra‐myocardial injection after heart failure,^13^, ^14^ TraceIT for cancer imaging and tissue spacing in radiation therapy,^15^ SpaceOAR for tissue spacing in radiation therapy,^16^, ^17^ and Bulkamid for transurethral injection to treat stress urinary incontinence^18^, ^19^), are approved by FDA or EMA. One of those products (SpaceOAR) has six current/active clinical trials. PAAG‐OA (Contura),^20^ an intra‐articular injection of poly(acrylamide) hydrogel, is still being evaluated in two new trials (NCT04179552 and NCT04045431) in Denmark for knee osteoarthritis. Gelstix, an injectable poly(acrylonitrile) hydrogel for treatment of lumbar intervertebral disc degeneration,^21^ is still being evaluated in NCT02763956 in Switzerland and the Netherlands. The remaining 66 current injectable hydrogel trials involve both new and previously approved products. Nearly half of those products are comprised of hyaluronic acid (31 unique trials, including the following products: MateRegen,^22^, ^23^ Hyalobarrier,^24^ HAmonyCa,^25^ KD Intra‐Articular gel, IPN‐21‐SENSE, Juvéderm,^26^ Sestylane, GAL 1906, Stylage, Auralya, I.SPACE, Belotero, Papilocare), or PEG (eight unique trials, including products Juveena, ReSpace, LifePearl,^27^ Instylla HES,^28^ and BioSentry BioSeal^29^). Noteworthy new products, which explore new hydrogel biomaterials or new hydrogel indications will be described in a later section.

### 4.3 Summary of non‐injectable hydrogel clinical trials

Our expanded clinical trial search yielded 139 new trials involving topical hydrogel application (81 trials) or non‐injectable hydrogel implants (58 trials). Representative trials are summarized in Table 2. Topical application of wound dressings, hydrogel contact lenses, and transdermal drug delivery systems are being tested for their treatment of several indications. Skin wounds treated with hydrogel therapeutics include radiation dermatitis, diabetic ulcers, and burns.^30^, ^31^ Biomaterials for topical dosage forms include synthetic carbomers, carboxymethylcellulose, chitosan, keratin, and poloxamer, in addition to the backbone polymers that are common across all hydrogel applications (i.e., HA, PEG, and silicone). Silicone continues to be especially common for application in hydrogel contact lenses,^32^, ^33^ with several clinical trials and approved products. Hydrogel implants are being applied for regenerative medicine, tissue augmentation, treatment of dental/periodontal disease, and prevention of postsurgical adhesions. Backbone hydrogel materials included a variety of biomaterials, including silicone hydrogels, non‐HA polysaccharide hydrogels, and ECM hydrogels, which are uncommon backbone polymers for both injectable hydrogels and current approved products. Six gel stent trials (XEN stent)^34^ are currently evaluating outcomes in the treatment of glaucoma.

**TABLE 2:** Summary of representative recruiting, enrolling, active clinical trials involving topical hydrogels, hydrogel contact lenses, and hydrogel implants.

Name (Company)	Indication	Hydrogel formulation	Drug delivered?	NCT number (Trial Phase)
Representative products—hydrogel implants in clinical trial				
BEAR Implant (Miach Orthopedics)	Anterior cruciate ligament injuries	Bovine collagen implant	No	NCT05398341 (not given)
Myriad Matrix, Myriad Morcells (Aroa Biosurgery Ltd.)	Abdominal Wound Dehiscence|Necrotizing Soft Tissue Infection|Lower Extremity Wound|Pilonidal Sinus|Anal Fistula|Hidradenitis Suppurativa|Pressure Injury	ECM implant	No	NCT05243966 (not given)
HANBIO BarriGel (HAN Biomedical Inc.)	Adhesion|Thyroid Diseases	Hyaluronic acid implant	No	NCT05036525 (NA)
BrachyGel (Brachy Foam, LLC)	Cervical cancer	PEG implant	No	NCT04499521 (NA)
DEXTENZA (Ocular Therapeutix, Inc.)	Vitreoretinal surgery, pain, inflammation	PEG implant	Yes, dexamethasone	NCT04371445 (Ph 4) and NCT04200651 (Ph 4)
AxoGuard (Axogen Corp.)	Symptomatic neuroma, chronic nerve pain	Porcine ECM implant	No	NCT03940963 (NA) and NCT04865679 (NA)
ESTYME (Symatese Aesthetics)	Bilateral breast augmentation	Silicone implant	No	NCT05336526 (NA)
Silimed (Silimed Industria de Implantes Ltda.)	Breast implant	Silicone implant	No	NCT03356132 (not given)
Motiva Implant (Motiva USA LLC)	Breast implant	Silicone implant	No	NCT03579901 (NA)
Representative products—topical hydrogels				
PTX‐022 (Palvella Therapeutics, Inc.)	Pachyonychia congenita, basal cell carcinomas in Gorlin syndrome patients	Topical carbomer 940	No	NCT05643872 (Ph 3) and NCT04893486 (Ph 2)
TCP‐25 (Xinnate AB)	Blister skin wound	Topical hydroxyethylcellulose	No	NCT05378997 (Ph 1)
Catasyn (Synedgen, Inc.)	Superficial partial thickness burn	Topical hydroxypropylcellulose	No	NCT04601532 (Ph 4)
StrataMGT (Strataphama AG)	Genitourinary syndrome of menopause	Topical silicone	No	NCT05672901 (NA)
StrataXRT (Stratapharma AG)	Radiation dermatitis	Topical silicone	No	NCT05553392 (NA)
Suprasorb (Lohmann & Rauscher)	Ulcer	Topical alginate	No	NCT05646121 (not given)
RadiaAce (AceTech)	Breast cancer radiation dermatitis	Topical acemannan	No	NCT04481802 (NA)
QY211 (E‐nitiate Biopharmaceuticals Co., Ltd.	Atopic dermatitis	Topical carbomer 947	No	NCT05843422 (Ph 1)
NanoDOX (NanoSHIFT LLC)	Wounds and injury	Topical carboxymethylcellulose	Yes, doxycycline	NCT05411484 (Ph 2)
NanoSALV (NanoTess, Inc.)	Ulcer	Topical cellulose	No	NCT05619237 (NA)
SynePure (Synedgen, Inc.)	Burn wounds	Topical chitosan	Yes, silver sulfadiazine	NCT05877638 (NA)
TTAX01 (Tissue Tech, Inc.)	Diabetic foot infection, non‐healing wound	Topical decellularized human placental umbilical cord tissue	No	NCT04450693 (Ph 3) and NCT04176120 (Ph 3)
Restrata, Apligraf (Acera Surgical, Inc.)	Diabetic foot ulcer, venous leg ulcer	Topical electrospun ECM	No	NCT04927702 (NA)
Juvia (FEMPHARMA Kft.)	Vaginal yeast infections	Topical hydroxyethylcellulose	Yes, zinc	NCT05895162 (NA)
KeraStat (KeraNetics, LLC)	Partial‐thickness burn	Topical keratin	No	NCT03564795 (NA)
BIAKOS (SeranaGropu, Inc.)	Wound of skin	Topical poloxamer407	Yes, polyaminopropyl biguanide	NCT05107050 (NA)
Representative product—hydrogel stent				
XEN45 (Allergan/AbbVie)	Open‐angle glaucoma	Porcine collagen‐derived gelatin hydrogel stent	No	NCT05411198 (Ph 3)
Representative product—hydrogel coil				
HydroSoft (Microvention‐Terumo, Inc.)	Ruptured aneurysm	Vinyl polymer hydrogel coil	No	NCT03252314 (not given)

*Note*: Trials are organized first by material form, then biomaterial composition.

### 4.4 New hydrogel biomaterials in active clinical trials

Our analysis identified three noteworthy categories of new hydrogel clinical trials, which are summarized in Table 3. The grouping includes five clinical trials (NCT04379700, NCT04951479, NCT05268718, NCT04595266, and NCT05051332) which are evaluating some of the first hydrogel nanogel or hydrogel microsphere suspensions for the treatment of diverse indications (e.g., osteoarthritis, ulcerative keratitis, metastatic cancer, and cartilage degeneration). The second are nine active trials involving new hydrogel biologic or hydrogel‐cell combination products (Products: SygeLIX, TumoCure, BioVAT, Placental Mesenchymal Stem Cells on Dural ECM, ALLO‐ACS‐DFU, DBI‐001, DBI‐002, TTAX01, and ArthoFLEX). The final grouping highlights clinical trials evaluating new hydrogel materials, which have been designed to treat indications such as chronic rhinosinusitis, ruptured aneurysm, bacterial vaginoses, periodontal disease, bladder cancer, and more.

**TABLE 3:** Summary of recruiting, enrolling, active clinical trials using novel hydrogel biomaterials or dosage forms.

Name (sponsor company or university)	Indication	Route of administration	Hydrogel formulation	Drug delivered	ClinicalTrials.gov identifier (trial phase)
First nanogel/microgel based delivery systems as combination products					
Embozene microspheres (NYU Langone Health)	Osteoarthritis	Intraarticular injection	Polyzene‐F microspheres	No	NCT04379700 (NA)
OptiSphere (University of Pittsburgh)	Knee osteoarthritis	Intraarterial injection	Microspheres (unknown material)	No	NCT04951479 (NA)
Hollow gold and silver alloy cuprous oxide shell nano‐shell hydrogel (Zhejang University)	Ulcerative keratitis, antibiotic resistance, photothermal therapy	Topical application	Nanogel suspension	No	NCT05268718 (early Ph I)
LifePearl (Grupo Espanol Multidisciplinario del Cancer Digestivo)	Metastatic colorectal cancer, liver metastasis	Intraarterial injection	PEG microgel suspension	Yes, irinotecan	NCT04595266 (Ph II)
CartiLife (Biosolution Co., Ltd.)	Articular cartilage defect, articular cartilage degeneration	Intraarticular injection	Fibrin microsphere	Yes, chondrocytes	NCT05051332 (Ph III)
Gel biologics + cell combination products					
SygeLIX‐F, SygeLIX‐G (TBF Genie Tissulaire)	Anal fistula	Implant via internal orifice	Bulk Wharton's Jelly gel plug	Yes, stem cells	NCT05638139 (Ph I)
TumoCure (IntraGel Therapeutics)	Head and neck cancer	Intratumoral injection	Bulk polymeric gel	Yes, cisplatin	NCT05200650 (Ph I)
BioVAT (University Medical Center Coettingen)	Heart failure	Implant in ventricular myocardium	Bulk collagen hydrogel with iPSC‐derived cardiomyocytes	Yes, cardiomyocytes	NCT04396899 (Ph I, Ph II)
Placental mesenchymal stem cells on dural ECM (California Institute for Regenerative Medicine)	Myelomeningocele	Topical application	Bulk dural graft ECM hydrogel	Yes, mesenchymal stem cells	NCT04652908 (Ph I, Ph II)
ALLO‐ACS‐DFU (Anterogen Co., Ltd.)	Diabetic foot ulcer	Topical application	Hydrogel SHEET dressing	Yes, allogenic mesenchymal stem cells	NCT03754465 (Ph II)
DBI‐001, DBI‐002 (DermBiont, Inc.)	Tinea pedis	Topical application	Unknown aqueous gel	Yes, microbe	NCT05493488 (Ph II)
TTAX01 (Tissue Tech Inc.)	Non‐healing diabetic foot ulcer	Topical application	Decellularized human placental umbilical cord tissue	No	NCT04450693 (Ph III)
TTAX01 (Tissue Tech Inc.)	Non‐healing diabetic foot ulcer	Topical application	Decellularized human placental umbilical cord tissue	No	NCT04176120 (Ph III)
ArthoFLEX ECM scaffold (Cleveland Clinic)	Rotator cuff tear	Implant via arthroscopic procedure	Bulk ECM hydrogel	No	NCT03551509 (Ph IV)
New polymers/materials (fewer than two trials and no approved products)					
Chitodex Gel (St. Louis University)	Chronic rhinosinusitis	Topical application in nasal cavity	Chitosan and dextran	No	NCT05083741 (not given)
CartRevive (Hy2Care BV, Avania, UMC Utrecht)	Cartilage damage	Intraoperative injection in knee	Dextran + hyaluronic acid	No	NCT05186935 (NA)
Multi‐Gyn ActiGel (BioClin BV, Avania, Karo Pharma AB)	Bacterial vaginoses	Topical application to vagina	Galactoarabinan polyglucoronic acid crosspolymer	No	NCT04807842 (NA)
Aquacryl (Ain Shams Maternity Hospital)	Early abortion	Implant in cervical canal	Poly(acrylonitrile)	No	NCT05147857 (NA)
Condrotide® (Mastelli S.r.I, Latis S.r.I.)	Meniscus tear, meniscus lesion	Intraarticular and intrameniscal injection	Polynucleotide	No	NCT05322005 (NA)
Plenhyage® (I.R.A. Istituto Ricerche Applicate S.p.A.|Opera CRO, a TIGERMED Group Company)	Cicatrix, lipodystrophy, plaque	Intradermal injection	Polynucleotides (PDRN) of animal origin (fish)	No	NCT05239117 (NA)
Emdogain® FL (Aristotle University of Thessaloniki)	Periodontal Disease, AVDC Stage 3 and 4	Periodontal implant	Propylene glycol alginate and Porcine enamel matrix derivative	No	NCT05541614 (NA)
SMI‐01 (Sofregen Medical, Inc., Symbio, LLC).	Nasolabial fold, cheek augmentation	Intradermal injection	Silk	No	NCT04534660 (NA)
Particulate xenograft erythropoietin gel (Ain Shams University)	Periodontal bone loss	Periodontal implant	Erythropoietin and carboxymethylcellulose	Yes, particulate xenograft	NCT05360511 (Early Ph I)
PRO‐165 (Laboratorios Sophia S.A de C.V.	Dry eye syndromes	Topical application to eye	Chondroitin sulfate and hyaluronic acid	No	NCT03697876 (Ph I)
UGN‐102 (UroGen Pharma Ltd.)	Bladder cancer, urothelial carcinoma	Intravesicular injection (into bladder via catheter)	PEO/PPO triblock copolymer	Yes, mitomycin	NCT05243550 (Ph III)
UGN‐102 (UroGen Pharma Ltd.)	Bladder cancer, urothelial carcinoma	Intravesicular injection (into bladder via catheter)	PEO/PPO triblock copolymer	Yes, mitomycin	NCT05136898 (Ph III)
Hydrotac (Centre Francois Baclesse, Ministry of Health, France)	Head and neck cancer	Topical application to skin	Polyurethane	No	NCT01520701 (Ph III)
Chitosan‐based erythropoietin gel (Ain Shams University)	Erythropoietin recession	Topical application to gingiva	Chitosan and beta glycerophosphate	Yes, erythropoetin	NCT05683782 (Ph 4)

*Note*: Trials are organized by their category of technical novelty.

### 4.5 New indications for hydrogel therapy in active clinical trial

Our research identified a final set of noteworthy active clinical trials, where hydrogel therapeutics (approved and new) are being evaluated for new indications. These clinical trials are summarized in Table 4. These indications, which non‐exhaustively include osteoarthritis of joints other than the knee, esophageal and colorectal diseases, surgically acquired infection, nerve pain, and infertility currently lack approved hydrogel products. Most of these trials are evaluating common backbone polymers (i.e., HA, PEG, carbomer, and ECM), formulated into first‐in‐class hydrogel products. Some of these products (e.g., ADAM system, a male birth control device developed by Contraline Inc.)^35^ propose truly emerging therapeutics, as they offer a solution for a medical problem or disease indications with no approved biomaterial or pharmacological products.

**TABLE 4:** Summary of recruiting, enrolling, and active clinical trials proposing new disease indications for hydrogel‐based dosage forms, delivery systems, or therapeutics.

Name (sponsor company or university)	Indication	Route of administration	Hydrogel formulation	Drug delivered	ClinicalTrials.gov Identifier (Trial Phase)
KD Intra‐Articular® gel (Procare Health Iberia S.L.)	Hip, thumb, or ankle osteoarthritis	Intraarticular injection	Hyaluronic acid	No	NCT05275244 (not given)
Purastat (AdventHealth)	Esophageal stricture	Topical application to GI lesions via endoscope	Synthetic peptide	No	NCT05581173 (not given)
Bridge‐Enhanced ACL Restoration (BEAR®) (Miach Orthopedics)	Anterior cruciate ligament injuries	Surgical implant	Bovine collagen	No	NCT05398341 (not given)
ORISE gel (Portsmouth Hospitals NHS Trust, etc.)	Colorectal polyp	Submucosal injection during endoscopy	Poloxamer	No	NCT04886609 (not given)
Condrotide (Mastelli S.r.I, Latis S.r.I.)	Meniscus tear, meniscus lesion	Intraarticular and intrameniscal injection	Polynucleotide	No	NCT05322005 (NA)
DAC‐AE (Centre Hospitalier Universitaire de Saint Etienne, Ministry of Health, France)	Hip prosthesis infection	Surgical implant	Hyaluronic acid and poly(lactic acid)	Yes, antibiotic	NCT04251377 (NA)
DAC, Novagenit SRL (Centre Hospitalier Universitaire de Saint Etienne, Ministry of Health, France)	Hip prosthesis infection	Surgical implant	Hyaluronic acid, poly‐lactic acid	Yes, antibiotic	NCT04251377 (NA)
Papilocare (Procare Health Iberia S.L., Adkoma Health Research)	Human papilloma virus, human papilloma virus infection, cervix lesion	Intravaginal injection via cannula	Hyaluronic acid, beta glucans	No	NCT04199078 (NA)
Axoguard Nerve Cap (Axogen Corp.)	Symptomatic neuroma, amputation, chronic nerve pain	Surgical implant	Porcine ECM	No	NCT04865679 (NA)
Axoguard Nerve Cap (Axogen Corp.)	Symptomatic neuroma, Morton's neuroma, chronic nerve pain	Surgical implant	Porcine ECM	No	NCT03940963 (NA)
GelStix (Ospedale Regionale di Lugano, Rijnstate Ziekenhuis, Arnhem, the Netherlands)	Degeneration of lumbar intervertebral disc	Intradiscal injection	Hydrolysed poly(acrylonitrile)	No	NCT02763956 (NA)
MectaShield (Medacta International SA)	Arthritis, traumatic arthritis, avascular necrosis	Surgical implant	Unknown	Yes, antibiotic	NCT05679232 (NA)
JointRep (Oligo Medic Pty Ltd. Mobius Medical Pty Ltd.)	Articular cartilage defect	Intraarticular injection	Chitosan	No	NCT04840147 (NA)
ADAM system (Contraline Inc.)	Azoospermia, oligospermia	Injection into vas deferens	Unknown	No	NCT05134428 (NA)
MateRegen (Chinese University of Hong Hong)	First trimester abortion, surgical abortion, miscarriage with afibrinogenemia	Intrauterine injection	Hyaluronic acid	No	NCT05360186 (NA)
Hyalobarrier Gel (Ghent University Hospital, etc.)	Infertility, uterine polyp uterine myoma, uterine adhesion, hysteroscopy, uterine septum	Intrauterine injection	Hyaluronic acid	No	NCT03880435 (NA)
Hyalobarrier Gel (Ghent University Hospital)	Myoma	Intrauterine injection	Hyaluronic acid	No	NCT05683041 (NA)
Hyalobarrier Gel (SciVision Biotech Inc)	Tissue adhesion in gynecologic surgery	Intrapelvic implant	Hyaluronic acid	No	NCT04063085 (NA)
Juvia (Fempharma Kft.)	Vaginal yeast infections	Topical application to vagina	Hydroxyethylcellulose	Yes, zinc	NCT05895162 (NA)
StrataMGT (Stratpharma AG)	Genitourinary syndrome of menopause	Topical application to vagina	Silicone	No	NCT05672901 (NA)
VersaWrap (University of Colorado, Denver)	Distal radius fracture, tendon rupture	Surgical Implant	Hyaluronic acid and alginate	No	NCT04976335 (NA)
HANBIO BarriGel (HAN Biomedical Inc.)	Adhesion, thyroid diseases	Surgical Implant	Hyaluronic acid	No	NCT05036525 (NA)
Embrace Hydrogel Embolic System (HES) (Instylla, Inc.)	Arterial bleeding in solid organs and peripheral arteries	Intraarterial injection	PEG	No	NCT05364502 (NA)
Hydrocoil (Seoul National University Hospital, etc.)	Ruptured aneurysm, intracranial aneurysm	Intracranial implant in Aneurysm	Unknown	No	NCT04988503 (NA)
ATX01 (Algo Therapeutix)	Chemotherapy‐induced peripheral neuropathy	Transdermal delivery	Unknown	Yes, amitriptyline hydrochloride	NCT05593614 (Ph II)
Androgel (University Hospital, Clermont‐Ferrand)	Hypermetabolism in ICU	Transdermal delivery	Carbomer 980	Yes, testosterone	NCT03678233 (Ph II)
Androgel (VA Office of Research and Development)	Type 2 diabetes mellitus, hypogonadism	Transdermal delivery	Carbomer 980	Yes, testosterone	NCT03887936 (Ph IV)
Juveena (Rejoini Inc.)	Intrauterine adhesion	Intrauterine injection	PEG	No	NCT05394662 (Ph III)
FORTESTA (Endo Pharmaceuticals)	Male hypogonadism, hypogonadotropic hypogonadism	Transdermal delivery	Carbomer	Yes, testosterone	NCT04456296 (Ph IV)

*Note*: Trials are organized by phase.

## 5 CONCLUSION

Hydrogel materials are exciting candidates for therapeutics due to their ability to mimic native tissue environments and deliver a variety of therapeutic payloads. Our analysis indicates that hydrogels have been successfully translated as more than 100 medical products, for a variety of aesthetic, tissue repair, wound healing, tissue support, cancer, and ophthalmic applications. Emerging applications for therapeutic hydrogels include hydrogel coils for aneurysm treatment, hydrogel stents for glaucoma, new hydrogel wound dressings or patches for burns, diabetic ulcers, and radiation‐induced skin damage. New injectable hydrogels are being evaluated for application in pain management, regeneration of non‐musculoskeletal tissues, treatment of infection, female reproductive health, and male birth control. New non‐injectable hydrogels have been formulated as well, as coatings on medical implants, as bulk material implants, and in other forms. Depending on the nature of a new hydrogel device, its intended use, and its substantial equivalence to previously approved products, these new products may be classified as a class I, II, or III device. It is noteworthy that current hydrogel devices have received a range of classifications from the FDA, from silicone hydrogels and hydrogel burn dressings (class I device), to injectable hydrogel spacers for rectal radiotherapy (class II device) and wound dressings containing drugs and biologics (currently unclassified and pre‐amendment). While these translational challenges to hydrogels and hydrogel combination products persist, the presented data indicate that alternate hydrogel dosage forms (e.g., topical dressings, implants, stents, coils, microspheres, and nanogel suspensions) and new materials (e.g., peptide gels, nucleotide gels, and new synthetic polymers) are enabling a wide range of therapeutic hydrogel applications.

## AUTHOR CONTRIBUTIONS

**John R. Clegg:** Conceptualization; formal analysis; methodology; writing – original draft; writing – review and editing. **Kolade Adebowale:** Conceptualization; formal analysis; methodology; writing – original draft; writing – review and editing. **Zongmin Zhao:** Conceptualization; formal analysis; methodology; writing – original draft; writing – review and editing. **Samir Mitragotri:** Conceptualization; supervision; writing – review and editing.

### PEER REVIEW

The peer review history for this article is available at https://www.webofscience.com/api/gateway/wos/peer-review/10.1002/btm2.10680.

## Data Availability

All data are available in the main text.
